# Every fifth published metagenome is not available to science

**DOI:** 10.1371/journal.pbio.3000698

**Published:** 2020-04-03

**Authors:** Ester M. Eckert, Andrea Di Cesare, Diego Fontaneto, Thomas U. Berendonk, Helmut Bürgmann, Eddie Cytryn, Despo Fatta-Kassinos, Andrea Franzetti, D. G. Joakim Larsson, Célia M. Manaia, Amy Pruden, Andrew C. Singer, Nikolina Udikovic-Kolic, Gianluca Corno

**Affiliations:** 1 Molecular Ecology Group (MEG), Water Research Institute, National Research Council of Italy, Verbania Pallanza, Italy; 2 Technische Universität Dresden Institut für Hydrobiologie, Dresden, Germany; 3 Eawag, Swiss Federal Institute of Aquatic Science and Technology, Kastanienbaum, Switzerland; 4 Institute of Soil Water and Environmental Sciences, Volcani Center, Agricultural Research Organization, Rishon Lezion, Israel; 5 Department of Civil and Environmental Engineering and Nireas-International Water Research Center, University of Cyprus, Nicosia, Cyprus; 6 University of Milano Bicocca, Milan, Italy; 7 Centre for Antibiotic Resistance Research (CARe), University of Gothenburg, Gothenburg, Sweden; 8 Department of Infectious Diseases, Institute of Biomedicine, Sahlgrenska Academy, University of Gothenburg, Gothenburg, Sweden; 9 Universidade Católica Portuguesa, CBQF—Centro de Biotecnologia e Química Fina–Laboratório Associado, Escola Superior de Biotecnologia, Porto, Portugal; 10 Department of Civil & Environmental Engineering, Blacksburg, Virginia, United States of America; 11 UK Centre for Ecology & Hydrology, Wallingford, United Kingdom; 12 Division for Marine and Environmental Research, Rudjer Boskovic Institute, Zagreb, Croatia

## Abstract

Have you ever sought to use metagenomic DNA sequences reported in scientific publications? Were you successful? Here, we reveal that metagenomes from no fewer than 20% of the papers found in our literature search, published between 2016 and 2019, were not deposited in a repository or were simply inaccessible. The proportion of inaccessible data within the literature has been increasing year-on-year. Noncompliance with Open Data is best predicted by the scientific discipline of the journal. The number of citations, journal type (e.g., Open Access or subscription journals), and publisher are not good predictors of data accessibility. However, many publications in high–impact factor journals do display a higher likelihood of accessible metagenomic data sets. Twenty-first century science demands compliance with the ethical standard of data sharing of metagenomes and DNA sequence data more broadly. Data accessibility must become one of the routine and mandatory components of manuscript submissions—a requirement that should be applicable across the increasing number of disciplines using metagenomics. Compliance must be ensured and reinforced by funders, publishers, editors, reviewers, and, ultimately, the authors.

## Abstract 2

Have you ever sought to use metagenomic DNA sequences reported in scientific publications? Were you successful? This Essay reveals that every fifth published metagenome is not available to science, and asks whether data accessibility should be considered a mandatory component of manuscript submission.

## Drivers of open data

Science, as an ‘institution of organised criticism’ [1], progresses through the act of building on communal knowledge by ‘standing on the shoulders of giants’. Information sharing has been the pillar of scientific advancement ever since the first scientific journal, *Philosophical Transactions of the Royal Society*, in 1666. Open Data is the engine of science. High-throughput DNA sequence outputs are a relatively new form of data that offers exciting opportunities for hypothesis testing and retesting. Such data can best serve science if they adhere to 3 principles: transparency, reproducibility, and reusability [2]. GenBank of the National Center for Biotechnology Information (NCBI), Metagenomic Rapid Annotations using Subsystems Technology (MG-RAST), the European Nucleotide Archive (ENA), and the DNA Data Bank of Japan (DDBJ) are just a few examples of online repositories of DNA sequences. Several scientific achievements in recent years have relied on publicly available DNA data sets (reviewed by [3]), including assembled genomes from uncultured organisms [4], the discovery of the CRISPR-Cas systems [5], and unravelling the relationship between microbiomes or genetic features and specific diseases [6].

The benefits of Open Data to science are clear. It is proposed by many governments that the publishing of government-funded research data in a transparent, reproducible, and reusable format can ‘strengthen citizen engagement and yield new innovative businesses’ [7]. ‘Data can also be used in innovative ways that bring economic benefits to citizens and businesses by releasing untapped enterprise and entrepreneurship’ [8]. The leveraging of influenza virus (RNA) sequences [9] for the development of novel, more efficacious influenza vaccines [8] stands as one of a handful of clear-cut examples of the potential societal and economic benefits from Open Data. The emergence of the Severe Acute Respiratory Syndrome Coronavirus 2 (SARS-CoV-2) also clearly demonstrates the synergy that can come from ensuring Open Access to sequencing data—bringing rapid insights that can stem the spread of a potentially pandemic virus in real time [10,11].

## Current state of data availability

We screened all studies containing primary metagenomic data accessible through Clarivate Web of Science from the beginning of 2016 to March 2019 (see S1 Text) to quantify the current state of open science in studies that generate metagenomic data. We focused on metagenomic data such as shotgun metagenomics because they represent a rich and rapidly growing data set that can be very easily and fruitfully remined and repurposed for a wide range of hypothesis testing.

**Fig 1:**
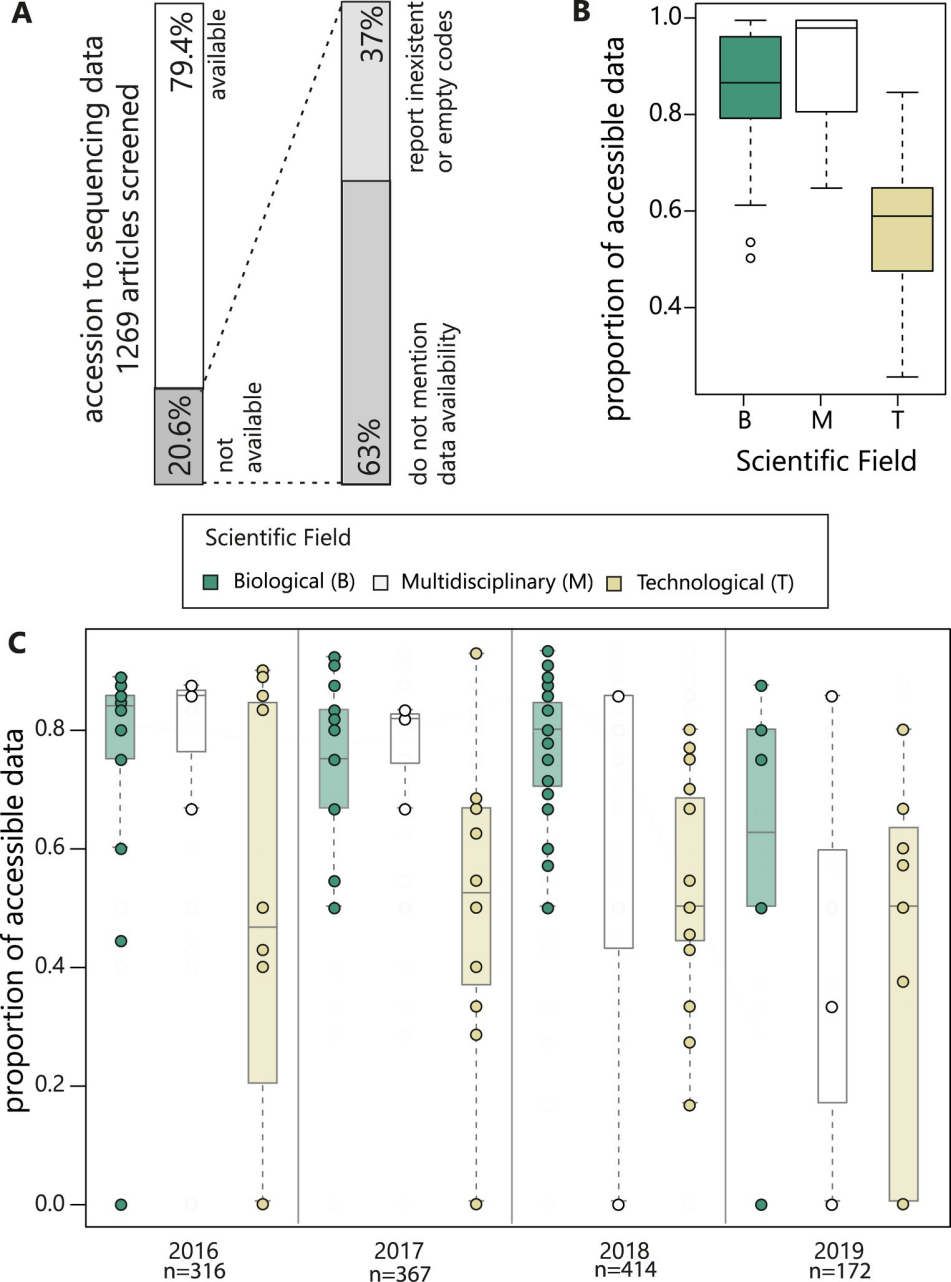
Main trends in accessibility of metagenomic data. (A) Proportion of papers with accessible metagenomic data and type of inaccessible data (grey) (B) in B, M, or T journals. (C) Temporal trends divided by B, M, and T journals. B, biological; M, multidisciplinary; T, technological.

Of the 1,269 publications we considered, 262 (20.6%) did not provide public access, either by not mentioning data availability (12.9%) or by reporting nonexistent or empty accession numbers (7.7%; Fig 1A). Conversely, 990 publications (78.0%) reported accession numbers or a link to the data. Five (0.4%) studies deposited sensitive data that require authorised access, and twelve (1%) stated that data are available from the authors upon request. Among the studies that offered no public access to data, only eight represented naïve mistakes, e.g., by erroneously reporting the metagenome submission code (SUB) that NCBI provides upon submission, which cannot be used to retrieve the data set, or with typos in the code preventing data accessibility.

Data set accessibility was significantly different across journals (generalised linear model [GLM], binomial: likelihood ratio chi-squared test [LR Chisq]_58_ = 266.9, *p* < 0.0001; S1 Data) and continued to decline with each passing year (LR Chisq_1_ = 4.9, *p* = 0.026). The proportion of unavailable published metagenomes increased (GLM, binomial: LR Chisq_1_ = 7.5, *p* = 0.006; S1 Fig) annually by 2% from 17% in 2016 (55 out of 316), 19% in 2017 (69 out of 367), and 21% in 2018 (87 out of 414). This trend continues into 2019 (up to March; 29%, or 49 out of 172). Accessibility of data was not correlated with the number of citations (time-regressed residual values S1 Fig) (LR Chisq_1_ = 3.8, *p* = 0.060).

Searching for correlates at the journal level, differences between disciplines—as defined by Clarivate Analytics (generalised linear mixed effects model [GLMEM], binomial: LR Chisq_10_ = 23.3, *p* = 0.0097)—were significant (S2 Fig). To better define the distribution of unavailable data across scientific disciplines, we assembled journals into 3 major categories (biological, multidisciplinary, and technological/applied sciences; see S1 Text, S2 Data). Data accessibility was significantly better for biological journals (with 13% of unavailable metagenomic data) and the multidisciplinary journals (10%) than for technological/applied sciences (43%) (Fig 1B) (GLM, binomial: LR Chisq_1_ = 5.9, *p* = 0.015; Fig 1C). We hypothesise that an increase in the proportion of technological/applied science papers using metagenomics can account for the overall temporal increase in noncompliance over time.

Overall, the proportion of papers with available metagenomes was significantly different between biological, multidisciplinary, and technological/applied science journals, regardless of Open Access, the age of the journal, and the publisher (Table 1). Regarding publishers, none of the publishing companies with at least 2 journals managed to achieve full compliance with data accessibilities (S1 Data), and the differences between publishers are only marginally significant (*p* = 0.0447; S3 Fig). This can be considered as a consequence of the huge difference in the data available between journals from the same publisher. For example, in the Nature Publishing Group, all journals share the same policies on data availability (https://www.nature.com/nature-research/editorial-policies/reporting-standards). Submission of metagenomic raw data to a community-endorsed, public repository is mandatory. Studies in the Nature Publishing Group journals *Nature* and *Nature Microbiology* all complied with Open Data, while others, such as *Scientific Reports*, revealed missing data for over 35% of the articles queried in this study.

**Table 1:** Potential drivers of the proportion of papers with accessible data for each journal.

Driver	LR Chisq	df	*p*	Effect
**B-M-T**	16.9	2	0.0002	-
**Number of papers**	10.8	1	0.0010	Negative
**Impact factor**	7.1	1	0.0075	Positive
**Publisher**	24.1	14	0.0447	-
**Open Access**	2.6	1	0.1094	-
**Age**	0.4	1	0.8378	Negative

Data are accounting for the following: the differences among biological, multidisciplinary, and technological journals (B-M-T), the impact factor (in log scale), the number of papers (in log scale), the publisher, Open Access, and the age (in log scale). Analysis of the deviance table from binomial GLM with the LR Chisq, df, *p*-value, and directionality of the effect for continuous variables.

**Abbreviations:** B-M-T, biological, multidisciplinary, and technological journal; df, degrees of freedom; GLM, generalised linear model; LR Chisq, likelihood ratio chi-squared test

Analysis of the impact factor reveals that the journals with the highest impact factor (Table 1 and S3 Fig) fully comply with data accessibility for metagenomes (e.g., *Nature*, *Nature Microbiology*, and *Science*). Journals publishing higher numbers of papers per year had a lower proportion of accessible data (S3 Fig and Table 1), with some exceptions: *Nature*, *Science*, *Peer J* (100% compliance), and *Nature Communications* (97%). Six ‘high volume’ journals correctly provided access to raw data for only 25%–50% of their published articles containing metagenomic data (S1 Data and S4 Fig).

## Action to ensure ‘open sequencing data’

Noncompliance with Open Data necessitates failures by the authors, journal managers, editors, and reviewers. Chronic failures to ensure Open Data should also be prioritised by funders, as there might be insufficient disincentives for noncompliance. Differences among journal policies (or lack thereof) and their ability to enforce such policies might explain some of these deficiencies. Technological/applied science journals are a rapidly growing publisher of studies containing metagenomic data and are currently the least likely publisher to comply with Open Data. It is clear that the best practice between disciplines is not being shared and that publishers and editors must work towards harmonising metagenomic submission requirements across disciplines and ensure compliance is obligatory.

On average, between 50 and 90 metagenome-containing articles per year are not available out of the 300 to 400 papers per year we screened, with an abrupt increase of 50 metagenomes out of 170 for the first months of 2019. Although the estimates of open metagenomic data compliance are not inclusive of all studies containing metagenomes, we feel this study does represent an unbiased survey of the literature. As such, we feel that this study should be used as a warning of a potentially growing problem, particularly in journals without a tradition of publishing studies with metagenomes and perhaps without access to the kind of reviewer base that holds Open Data as a priority.

Data availability reflects on the transparency of the scientific research and its reliability—compliance is a matter of scientific integrity and should be a requirement for all research that has public funding. Barriers to compliance with best practice need to be explored to ensure that complete Open Data is achievable. It should be noted that making data available is only the first step. Equally essential is the extent and accuracy to which associated metadata are provided, which are needed to put results into context and inform follow-up studies. Technical problems might also hamper accessibility to data sets. Of the studies that reported that they submitted their data to MG-RAST, we were unable to find 34.0%, meaning that one-third of the reported accession numbers for MG-RAST did not lead to sequence data (29 out of 85 articles). This happened only for 6.2% of the NCBI/ENA/DDBJ accession numbers (difference: Chisq_1_ = 76.7, *p* < 0.0001). Furthermore, sequencing data was the only data type analysed here, and the archiving of other types of primary data is much less standardised and common compared to nucleic acid data [12,13]. For phylogenies, for example, it has been estimated that 60% of the data are not available to science [14]. Guidelines for sequence submission are standard for most journals, but such standards might not be true for other primary data; an analysis of the guidelines for authors of biomedical journals showed that only 12% of journals required general data sharing for publications [15]. Furthermore, the analysis presented here quantifies those studies that reported an accession number or link to sequence data, but it does not verify the quality of the attached metadata. Dubious metadata might additionally hamper the usefulness of submitted sequences for others (i.e., specific details of sampling conditions, location, associated environmental conditions, i.e., aerobic versus anaerobic, healthy or sick individuals, etc. [16]) and thereby increase the fraction of de facto unavailable metagenomes to science.

Tax-payer–funded research must be open to the global research community. We acknowledge there are barriers to compliance and all stakeholders need to ensure that these barriers are quickly and methodically addressed. Publishers without a history of publishing studies with metagenomic data need to learn the best practices from other publishers with a long and successful track record of ensuring Open Data. Data accessibility should be an ethical standard met by all researchers.

## Supporting information

S1 Text Materials and methods. (DOCX)

S1 Data List of journals with scientific fields. Fields, IF, publisher, and number of published papers according to Clarivate Analytics; DOAJ; main group according to the distinction between biological, multidisciplinary, or technological papers. DOAJ, Directory of Open Access Journals; IF, impact factor.
(XLSX)

S2 Data The data set used for the analyses. It contains the 1,269 papers that passed the filtering steps of our literature survey on papers published between 2016 and 2019.
(XLSX)

S1 Fig Trend of accessibility of metagenomes over time. Data referrals for the year 2016 till March 2019.
(TIFF)

S2 Fig Difference in proportion of papers with accessible metagenomic data across scientific fields. Data collected according to Clarivate Web of Science.
(TIFF)

S3 Fig Differences between the proportion of data accessibility. Data related to the (A) publisher, (B) journal impact factor, and (C) number of papers published. Trendlines in (B) and (C) are from the GLMs cited in Table 1.
(TIFF)

S4 Fig Graphical depiction of multicollinearity between predictors used for statistical models. (TIFF)

## Abbreviations

B-M-T: biological, multidisciplinary, or technological journal
DDBJ: DNA Data Bank of Japan
df: degrees of freedom
ENA: European Nucleotide Archive
GLM: generalised linear model
GLMEM: generalised linear mixed effects model
LR Chisq: likelihood ratio chi-squared test
MG-RAST: Metagenomic Rapid Annotations using Subsystems Technology
NCBI: National Center for Biotechnology Information
SARS-CoV-2: Severe Acute Respiratory Syndrome Coronavirus 2
SUB: metagenome submission code
