# Supplementary material for: Every fifth published metagenome is not available to science
Source: PLoS Biol. 2020 Apr 3;18(4):e3000698. doi: 10.1371/journal.pbio.3000698 (PMC7159239; doi:10.1371/journal.pbio.3000698)
Supplement: S1 Text — (DOCX) [file pbio.3000698.s001.docx]

**Every fifth published metagenome is not available to science**

Ester M. Eckert, Andrea Di Cesare, Diego Fontaneto, Thomas U. Berendonk, Helmut Bürgmann, Eddie Cytryn, Despo Fatta-Kassinos, Andrea Franzetti, D. G. Joakim Larsson, Célia M. Manaia, Amy Pruden, Andrew C. Singer, Nikolina Udikovic-Kolic, Gianluca Corno.

**Ethics Statement:** No animals nor human participants were involved in the production an analyses of the data used for this article.

**Supporting Information File 1**

**Supplementary Methods**

**Methods:** In April 2019, a search through Clarivate Web of Science Core Collection was performed with the keyword “metagenom*”, selecting only the Science Citation Index Expanded journals, for the years from 2016 to March 2019. The Boolean search allowed us to retrieve any variation of the term (i.e., metagenome, metagenomics, etc.). The search resulted in a list of 3120 published papers. We then discarded the journals that only rarely publish metagenomic data, by setting an arbitrary threshold of at least 15 primary research papers (excluding reviews) between 2016 and March 2019. Some journals that publish sequence data might be missing from the list of journals analysed here, due to fact that the word *metagenom** was not explicitly mentioned in the abstract, keywords or title. A total of 59 journals passing the preliminary screening were retained in the list and we screened their papers. For the journals with more than an arbitrary threshold of 40 papers dealing with metagenomics, we limited the screening to a random selection of 40 papers from the list, for a total of 1707 papers over the span of all the analysed years. After reading the papers in detail we removed 438 papers that were out of scope/not relevant (e.g. using already published metagenomes), and retained 1269 papers that indeed produced primary metagenomic data.

We then created a dataset (S2 Data) where, for each of the 1269 papers, we included the DOI (DI field according to Clarivate), the year of publication (PY), the journal (SO), the number of citations (TC, normalised by time since publishing for the analyse [see below]), and marked whether the metagenomic data was available or not, the type of problem making it not available, the repository (if mentioned in the paper), and the direct link to the data (if available). For each link, we checked whether the link produced downloadable data or if it referred to an empty project. All entries were checked independently by at least three researchers to ensure reliability.

A metagenome was considered available when actual and working accession numbers were provided, or when the authors claimed that such data could be available upon request. A metagenome was considered ‘not available’ only when no mention of it was present in the paper or when the accession numbers, even if mentioned, did not exist or referred to empty projects.

We followed the division into scientific fields by Clarivate Web of Science for each journal; the dataset retained 11 of such fields (S1 Data). Subsequently, we grouped the journals into three main categories, depending on whether they were strictly biological (e.g. *Nature* *Microbiology*, *Molecular Ecology*, etc.), multidisciplinary (e.g. *Nature*, *PLoS ONE*, *Science*, *Scientific Reports*, etc.) or technological/applied (e.g. *Environment Science & Technology*, *Water Research*, etc.). For each journal we also reported the publishing company, whether it was listed in the Directory of Open Access Journals (DOAJ, https://doaj.org/), and the total number of papers for each journal across the three years used in the analyses (2016-2018, excluding the data from the first three months of 2019, since publishing within a year might not be evenly distributed over all months). We applied two different metrics of scientific relevance: the journal’s most recent Impact Factor in 2018 from Clarivate Analytics ISI as a proxy of current relevance, and the age of the journal as a proxy of historical relevance (S1 Data).

Generalised Linear Models (GLMs) and Generalised Linear Mixed Effects Models (GLMEMs) with binomial errors for proportion data and continuous explanatory variables with scaled values were used to test for differences [1] using R v3.3.3 [2]. GLMEMs were used in the analyses when a random factor needed to be included, for example the journal identity when some journals fell in more than one journal category, and were performed with R package *lme4* v1.1-21 [3]. All outputs of statistical tests are reported as Analysis of Deviance Table (Type II Wald chi-square tests) from R package *car* v3.0-4 [4]. To include the number of citations in the analyses, we preliminary performed a regression analysis, using the Generalised Additive Model (GAM) in R package *gam* v1.16.1 [5], of citations against year of publication for each paper, because more recent papers would have had less time to accumulate citations, and then used the residuals of such model to assess the effect of number of citations in the statistical models. For variables with an unlikely linear response, e.g. age of the journal, Impact Factor, and number of papers, we used a log transformation of the data [1]. In addition, chi-squared tests were used to test for differences in contingency tables to assess differences between online repositories for open access data. A preliminary test for lack of multi-collinearity between predictors was performed (S4 Fig).

1. M. J. Crawley, The R book. Wiley, Chichester UK (2012).
2. R Core Team, R: A language and environment for statistical computing. R Foundation for Statistical Computing, Vienna, Austria (2017).
3. D. Bates, M. Maechler, B. Bolker, S. Walker, Fitting Linear Mixed-Effects Models Using lme4. *Journal of Statistical Software* **67(1)**, 1-48 (2015).
4. J. Fox, S. Weisberg, An {R} Companion to Applied Regression, Third Edition. Thousand Oaks CA: Sage. URL: https://socialsciences.mcmaster.ca/jfox/Books/Companion/ (2019).
5. T. Hastie, gam: Generalized Additive Models. R package version 1.16.1. https://CRAN.R-project.org/package=gam (2019).
