# Supplementary figures and images for: Every fifth published metagenome is not available to science
Source: PLoS Biol. 2020 Apr 3;18(4):e3000698. doi: 10.1371/journal.pbio.3000698 (PMC7159239; doi:10.1371/journal.pbio.3000698)

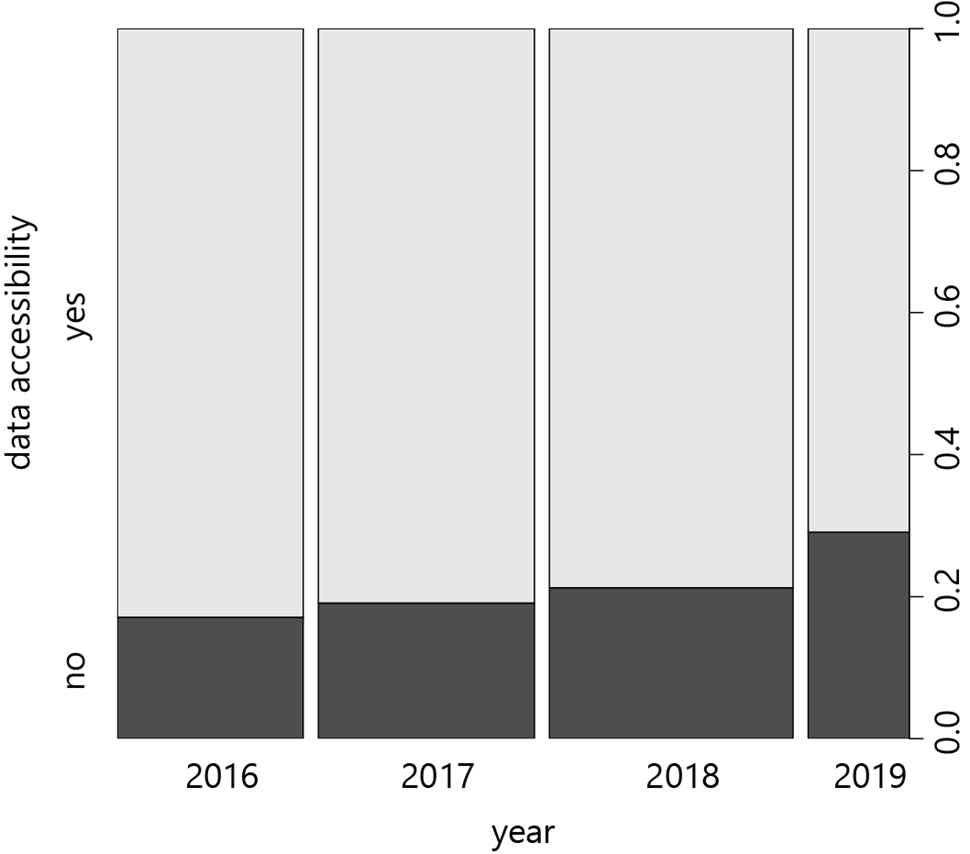

Supplement: S1 Fig — Data referrals for the year 2016 till March 2019. (TIFF) [file pbio.3000698.s004.tiff]

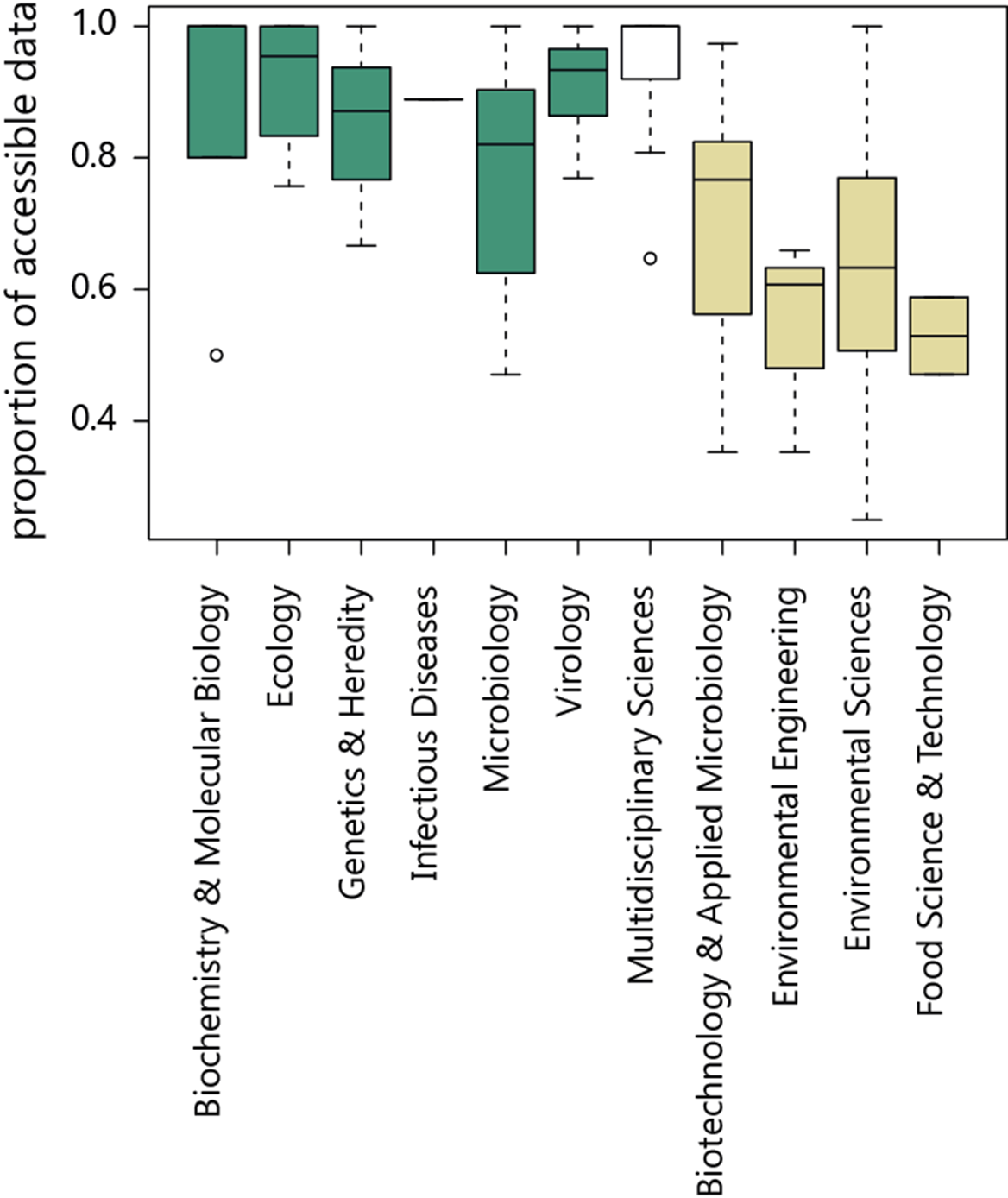

Supplement: S2 Fig — Data collected according to Clarivate Web of Science. (TIFF) [file pbio.3000698.s005.tiff]

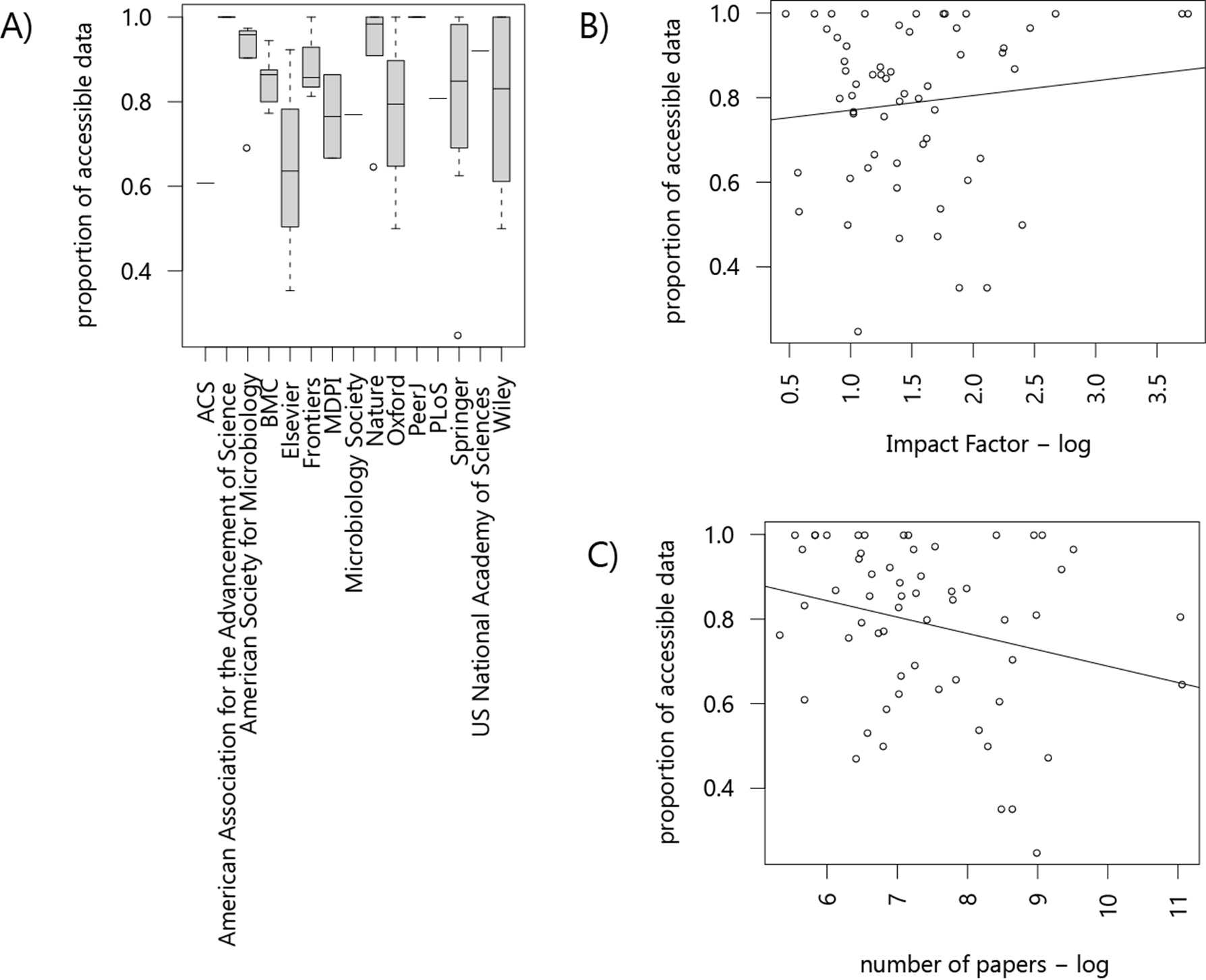

Supplement: S3 Fig — Data related to the (A) publisher, (B) journal impact factor, and (C) number of papers published. Trendlines in (B) and (C) are from the GLMs cited in Table 1. (TIFF) [file pbio.3000698.s006.tiff]

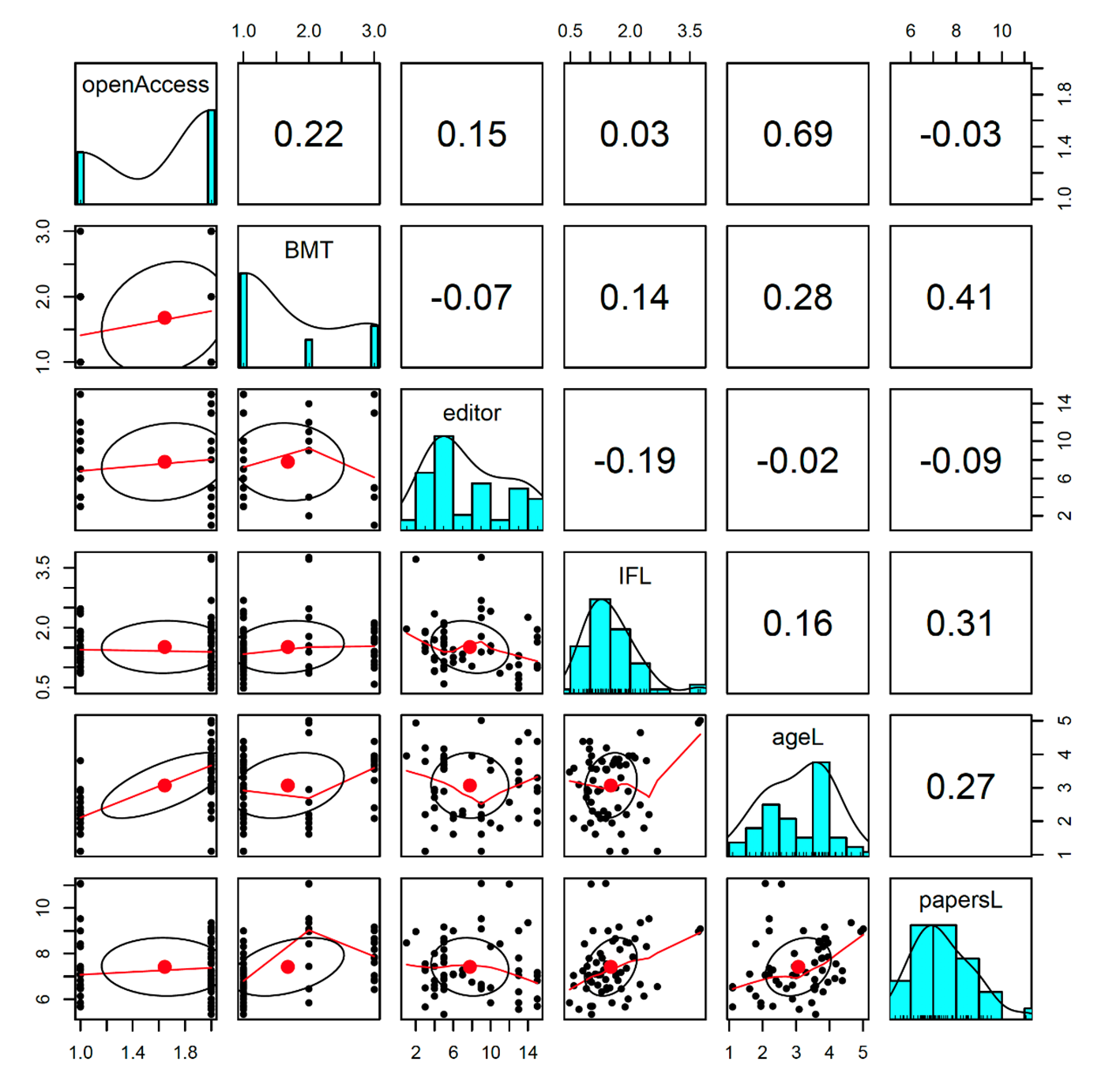

Supplement: S4 Fig — (TIFF) [file pbio.3000698.s007.tiff]
